# Chronic epidural intracranial actinomycosis: A rare case

**DOI:** 10.4103/0972-2327.56324

**Published:** 2009

**Authors:** S. K. Narayan, A. Swaroop, S. Jayanthi

**Affiliations:** Departments of Neurology, Jawaharlal Institute of Postgraduate Medical Education and Research, Pondicherry - 605 006, India; 1 Departments of Pathology, Jawaharlal Institute of Postgraduate Medical Education and Research, Pondicherry - 605 006, India

**Figure 1:**
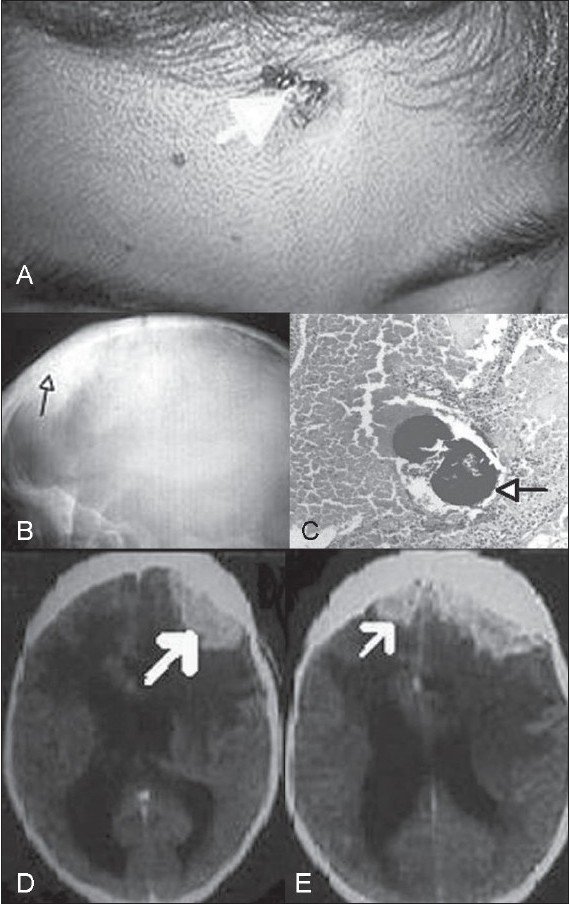
(A) Healing actinomycotic ulcer on the forehead; (B) Skull X-ray lateral view, showing sclerotic, thickened frontal vault (C) One deeply stained haematoxyphilic actinomyces colony amidst red blood cells and granulation tissue (H&E, ×150) (D and E) Contrast enhanced CT brain scan showing thickened cranial vault, brightly enhancing epidural mass with midline shift and oedema

A 13-year-old boy presented with a healing ulcer over the vertex, a healed ulcer above the right eye [Figure 1A], occasional fever, weight loss, diplopia, bilateral proptosis, lagophthalmos and bilateral mild facial palsy. In hospital, he developed left-sided focal seizures and symptoms and signs of raised intracranial pressure. The skull radiograph showed a thickened frontal vault, suggestive of chronic osteomyelitis [Figure 1B]. CT brain confirmed thickening of the skull vault over the right frontal region and revealed an epidural mass in the prefrontal region that was predominantly on the right side but crossed the midline and caused a mass effect, with midline shift to the left. Also present was massive white-matter edema, with effacement of the ipsilateral ventricle and enlargement of the contralateral one [Figure 1C]. A biopsy from the scalp lesion demonstrated granulation tissue, hematoxyphilic colonies, and gram positive filamentous rods, features that were diagnostic of actinomycosis [Figure 1D]. The boy recovered well withintravenous administration of crystalline penicillin and co-trimoxazole along with oral erythromycin for six weeks, followed by oral co-trimoxazole and erythromycin for six moths. The epidural mass and skull thickening, however, persisted.

Clinical features of chronic epidural lesions of the skull and spine can be subtle and treacherous. Signs, rather than the symptoms, of raised intracranial pressure often dominate. Spinal lesions may present earlier. Epidural mass lesions can be due to a variety of causes; these include (1) hematoma due to trauma, bleeding diathesis or venous sinus thrombosis; (2) malignant deposits from lymphoma, leukemia, multiple myeloma or chloroma; (3) chronic noninfectious granuloma due to sarcoidosis, eosinophilic granuloma, cholesteatoma, hypertrophic pachymeningitis, Wegener granulomatosis or cranial fascitis; (4) chronic infectious lesions, e.g., aspergillosis or tuberculosis (5); primary neoplasms like chondromas, chordoma, chondromyxoid fibroma, osteoblastoma, giant cell tumors of skull, Ewing sarcoma, congenital lipomatosis, histiocytosis and endometrial carcinoma.

Actinomycosis, a subacute or chronic granulomatous inflammatory disease, gives rise to suppuration, abscess formation and sinuses. The most common causative agent is Actinomycosis israeli, a gram-positive, acid-fast organism with some morphological resemblance to fungi.[1] Clinical forms include oro-cervico-facial (the commonest), thoracic, abdomino- pelvic, musculoskeletal and disseminated disease. The cerebral form is rare (< 5%) and may pose a diagnostic challenge, presenting as brain abscess (67%), meningitis/ meningoencephalitis (13%), actinomycetoma (7%), subdural empyema (6%) or epidural abscess (6%). Infection spreads by the hematogenous route from lung, oral cavity, abdomen or pelvis.[2,3] Dense fibrosis, a pathological hallmark of actinomycosis, is usually minimal in a cerebral lesion, while features characteristic of the disease at anatomic sites elsewhere (such as draining sinuses and sulfur granules) are not seen with epidural lesions. Diagnosis is usually confirmed by biopsy. Penicillin and Erythromycin are effective against actinomyces while a closely related species, nocardia is sensitive to co-trimoxazole.
